# Influence of ω-3 fatty acid eicosapentaenoic acid on IGF-1 and COX-2 gene expression in granulosa cells of PCOS women

**Published:** 2015-02

**Authors:** Vahideh Shahnazi, Mina Zaree, Mohammad Nouri, Mahzad Mehrzad-Sadaghiani, Shabnam Fayezi, Maryam Darabi, Sajjad Khani, Masoud Darabi

**Affiliations:** 1 *Women’s Reproductive Health Research Center, Al-zahra Hospital, Tabriz University of Medical Sciences, Tabriz, Iran.*; 2 *Department of Biochemistry and Clinical Laboratories, School of Medicine, Tabriz University of Medical Sciences, Tabriz, Iran.*; 3 *Students Research Committee, Infertility and Reproductive Health Research Center, Shahid Beheshti University of Medical Sciences, Tehran, Iran.*; 4 *Drug Applied Research Center of Tabriz University of Medical Sciences, Tabriz, Iran.*; 5 *Research Center for Pharmaceutical Nanotechnology, Tabriz University of Medical Sciences, Tabriz, Iran*

**Keywords:** *Eicosapentaenoic acid*, *Insulin-like growth factor 1*, *Cyclooxygenase 2*, *Granulosa cells*, *Polycystic ovary syndrome*

## Abstract

**Background:**

The omega-3 (ω-3) fatty acid eicosapentaenoic acid (EPA) is currently used in the clinic as a nutritional supplement to improve infertility, particularly in women with polycystic ovarian syndrome (PCOS).

**Objective:**

The present study was designed to investigate the effect of EPA on insulin-like growth factor 1 (IGF-1) and cyclooxygenase 2 (COX-2) gene expression in primary cultured granulosa cells from patients undergoing in vitro fertilization (IVF), and also to compare this effect with those in granulosa cells of PCOS patients.

**Materials and Methods:**

In this experimental study, human granulosa cells were isolated from follicular fluid of normal and PCOS women undergoing IVF by hyaluronidase digestions, followed by Percoll gradient centrifugation. Cells were cultured in vitro, exposed to a range of concentrations of the EPA (25-100 µM) for 24 hr, and investigated with respect to COX-2 and IGF-1 gene expression by real time-PCR.

**Results:**

In both groups, all doses of the EPA significantly induced IGF-1 mRNA gene expression compared to the untreated control. High doses of EPA in the presence of recombinant (r) FSH produced a stimulatory effect on IGF-1 and a suppressive effect (p=0.01) on the COX-2 gene expression, which were more pronounced in granulosa cells from PCOS patients.

**Conclusion:**

EPA affect diversely the gene expression of IGF-1 and COX-2 in granulosa cells, which were more pronounced in PCOS compared to control. These findings represent the possible underlying molecular mechanisms for the positive impact of the ω-3 fatty acids on reproduction, especially in patients with PCOS.

## Introduction

Granulosa cells are in close contact with oocytes which provide structural and metabolic support for oocytes. Abnormal granulosa cells function is related to infertility condition, such as ovulatory dysfunction associated with polycystic ovarian syndrome (PCOS) (1). PCOS is the most commonly occurring cause of female infertility (2). In PCOS there is an imbalance of sex hormones, which may lead to ovarian cysts and irregular or absent menstrual cycle. These complications have been mainly attributed to the suppression of the follicle stimulating hormone (FSH) secretion by an excess androgen produced from the ovary. Early follicular growth leads to attenuated FSH response and the premature luteinization of granulosa cells. In turn, the development of the dominant follicle is disrupted, which is followed by cystic follicular arrest (3). 

The cyclooxygenase 2 (COX-2), encoded by the Ptgs2 gene, is the rate-limiting enzyme in the synthesis of prostaglandins, such as prostaglandin E_2_ (PGE_2_). In ovarian granulosa cells, COX-2 is induced by gonadotropins during early follicle development. The timely expression of COX-2 prior to follicle rupture in granulosa cells plays a critical role in ovulation. The COX-2 knockout mice have impaired ovulation, indicating that these genes control ovulation and cumulus expansion (4). The follicular arrest of PCOS has also been characterized by the lack of in vivo FSH‐induced folliculogenesis and proliferation of granulosa cells (5). Normal response to the pre-ovulatory signal from gonadotropins, including luteinization and expansion of cumulus-oocyte complex, was dependent on COX-2 expression (6). 

Thus, COX-2 has been suggested as a marker of follicular commitment to ovulation during ovarian stimulation (7). PCOS is often associated with insulin resistance, and insulin-sensitizing agents are being used as treatment (8). Insulin-like growth factor 1 (IGF-1) is beneficial to insulin sensitivity and shares many signaling components and cellular responses with insulin. IGF-1 is expressed in the granulosa cells and augments the proliferating effect of FSH on granulosa cells (9, 10). The IGF-1 may interact with COX-2 and thereby contribute to the regulation of ovarian function (11, 12). Omega-3 (ω-3) fatty acids are known as important fatty acids in immune regulation, insulin sensitivity, cellular differentiation and ovulation. ω-3 fatty acids reduce the synthesis of prostaglandins through competitive inhibition of COX-2, as well as competing with arachidonic acid as the substrate for COX-2 (13). 

Previous studies have reported differential effects of ω-3 fatty acids on IGF-1 and COX-2 expression in several cell types (13-15). However, the effect of ω-3 fatty acids on granulosa cells with regard to the expression or co-expression of IGF-1 and COX-2 is not known. Several epidemiological reports suggested a possible benefit of ω-3 fatty acids on PCOS. In particular, eicosapentaenoic acid (EPA), a long-chain ω-3 fatty acid, has been implicated as a protective agent in cancer, atherosclerosis and inflammation. Despite the increasing clinical use, the mechanism by which EPA exerts its effects is not yet clearly known. The aim of the present study was to investigate the effects of EPA on IGF-1 and COX-2 gene expression in cultured granulosa cells from patients undergoing in vitro fertilization (IVF), and also to compare these effects with those in granulosa cells of PCOS patients.

## Materials and methods

This experimental study was carried out from July 2011 to September 2013 at Tabriz University of Medical Sciences. The study protocol was approved by the Ethics Committee of Tabriz University of Medical Sciences. All patients gave written informed consent and their confidentiality and anonymity were protected.

**Cell culture**

Primary human granulosa cells were obtained from a patient population scheduled for IVF at Alzahra Hospital in Tabriz. PCOS were defined as the presence of 12 or more follicles measuring 2-9 mm with clinical (a Ferriman-Gallwey score >7) and/or biochemical hyperandrogenism (total testosterone >3 nmol/l) (16). Inclusion criteria were no alcohol consumption and no smoking habit. Uterus abnormalities, endometriosis, anovulation, positive history of endocrine disease and inflammatory disorders such as thyroid and adrenal disorders, hormonal treatment, and immune system defect were considered as exclusion criteria in this study. Control group included age- (27.62±4.14 years) and BMI- (25.11±2.57 kg/m^2^) matched with no evidence of hyperandrogenemia or menstrual irregularities. 

All patients underwent a standard infertility evaluation, including hormonal testing and assessment of the uterus and fallopian tubes by means of hysterosalpingography. Patients underwent a long GnRH agonist (decapeptyl; Debio Pharm, Geneva, Switzerland) /FSH-long down regulation protocol as described previously by us (17). Granulosa cells were isolated from aspirated follicular fluid by hyaluronidase digestions, followed by Percoll gradient centrifugation (18). 

Three sets of experiments with both PCOS and control groups were performed. Granulosa cells were pooled because the number of cells from follicles was insufficient to perform individualized culture. In the experiments, each group composed of granulosa cells pooled from 5 women. In total, granulosa cells were isolated and pooled from 15 PCOS and 15 control women of reproductive age. The granulosa cells were counted with a homocytometer, and approximately 1×10^6^ cells were plated in a 12-well culture plate containing DMEM/F12 medium supplemented with 10% FBS, 100 IU/ml penicillin, and 100 µg/ml streptomycin, for 24 hr. Cells were maintained at 37^o^C in 5% CO_2_ in a humidified incubator. EPA (Sigma, St. Louis, MO) was conjugated with bovine serum albumin (BSA) fatty acid-free (Sigma, St. Louis, MO) before treatment (19). Granulosa cells, after serum starvation overnight, were treated with indicated concentrations of EPA (25-100 µM) for 24 hr, both either with or without pretreatment with recombinant (r) FSH (100 ng/mL).

**Real-time PCR analysis**

Total RNA was isolated using RNX-Plus according to the instructions of the manufacturer. RNA pellets were ethanol-precipitated, washed, and resuspended in sterile ribonuclease-free water. Two µg of total RNA were reverse transcribed into cDNA using SuperScript II reverse transcriptase (Life Technologies, Carlsbad, CA). Real-time PCR was carried out using the fluorescent dye SYBR-Green and a Bio-Rad CFX real-time PCR system (BioRad Co, CA). The primers and conditions used for qPCR of IGF-1, COX-2 and GAPDH (as internal control) genes were as described previously (20, 21). Samples were assayed in duplicates. The amount of specific PCR products was normalized to the GAPDH mRNA content, and quantities were expressed as an x-fold difference relative to a control.

**Statistical analysis**

Values were presented as mean±SD of 3 separate experiments done in duplicate. Statistically significant differences in mean values between groups were assessed by t-tests. Analysis of variance test were used for comparing the group means. Calculation of significance between groups was done according to analysis of variance (ANOVA) with post hoc Tukey’s tests for multiple comparisons, and a p<0.05 was considered statistically significant.

## Results

**Figure 1:**
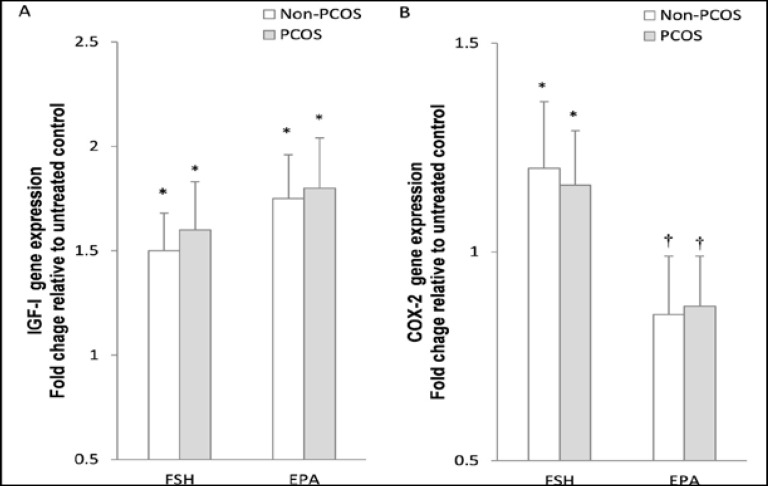
Effect of the rFSH and eicosapentaenoic acid (EPA) incubation on mRNA expression of IGF-1 and COX-2. Granulosa cells, after serum starvation, were incubated for 24 hr±100 ng/mL rFSH or 50 μmol/L EPA. Cell lysates were prepared and analyzed by real-time PCR for genes expression levels. Expression of IGF-1 (A) and COX-2 (B) in each lysate were normalized to the amount of GAPDH and represented as fold of untreated control. The mean±SD of three independent experiments with cells pooled from 5 women per group. * p<0.05 vs. untreated control. † p<0.05 vs. non-PCOS.

**Figure 2:**
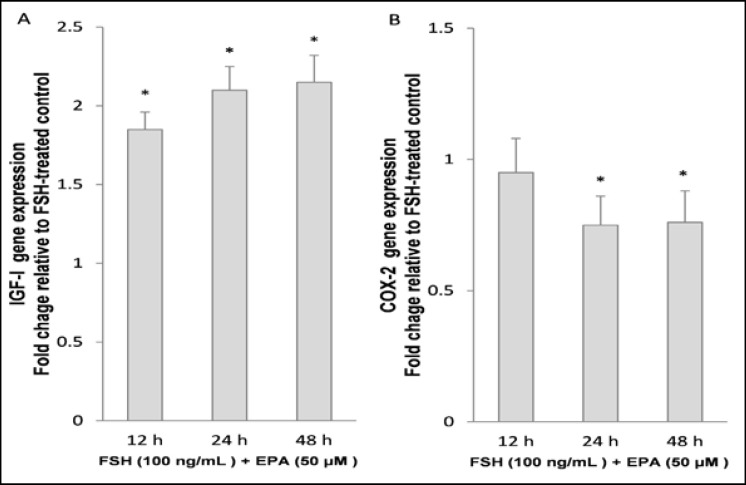
Effect of the eicosapentaenoic acid (EPA) incubation time on mRNA expression of IGF-1 and COX-2. Granulosa cells, after serum starvation, were incubated in 100 ng/mL rFSH alone or in combination with 50 μmol/L EPA for 12hr, 24hr and 48hr. Cell lysates were prepared and analyzed by real-time PCR for genes expression levels. Expression of IGF-1 (A) and COX-2 (B) in each lysate were normalized to the amount of GAPDH and represented as fold of rFSH-treated control. The mean ± SD of three independent experiments with cells pooled from 5 women per group. * p<0.05 vs. rFSH-treated control and 12hr incubation, respectively.

To determine the effect of rFSH stimulation on IGF-1 and COX-2 expression, granulosa cells were treated with rFSH. Both IGF-1 and COX-2 showed significant increases in mRNA levels (p=0.01, Figure 2), which were comparable between the PCOS and non-PCOS groups (p=0.01). Similarly, incubation with EPA alone resulted in comparable up-regulation of IGF-1 expression (>1.5 -fold; p=0.004) in granulosa cells from control and PCOS patients. However, a significant down regulation was observed for COX-2 expression in EPA-treated cells (Figure 1).

Comparison of control rFSH with the combined rFSH-EPA condition showed a similar, but more intense, response compared to the EPA alone. To optimize the assay, cultured granulosa cells from non-PCOS women were incubated with the 50 μmol/L EPA and the incubation time ranged from 12-48 hr. Both IGF-1 and COX-2 showed significant changes compared to control following EPA treatment, measured at 24h. These changes were 2.1±0.11 fold increase in IGF-1 and a 0.75-fold decrease in COX-2. However, prolonging the incubation time to 48h produced no further changes in the levels of both mRNAs (Figure 2). In the next series of experiments, three doses of EPA (0-100 μM) were tested in the presence of rFSH. Treatment of granulosa cells with 50 and 100 μM doses of the EPA significantly increased IGF-1 mRNA gene expression compared to the control rFSH alone condition (p=0.02). IGF-1 displayed a larger fold change in the PCOS group than in the non-PCOS group. The magnitude of this difference between non-PCOS and PCOS was more pronounced at the higher doses of EPA (e.g., 1.15-fold at 25 µmol vs. 1.29-fold at 100 μM; p=0.01). Moreover, it was identified that the expression level of COX-2 was also influenced by the higher doses of EPA in the PCOS granulosa cells as compared to the control. The combination of high doses of EPA in the presence of rFSH produced a relatively strong suppressive effect on the COX-2 gene expression in the PCOS granulosa cells as compared to the control non-PCOS (0.61-fold vs. 0.73, p=0.01; Figure 4).

**Figure 3:**
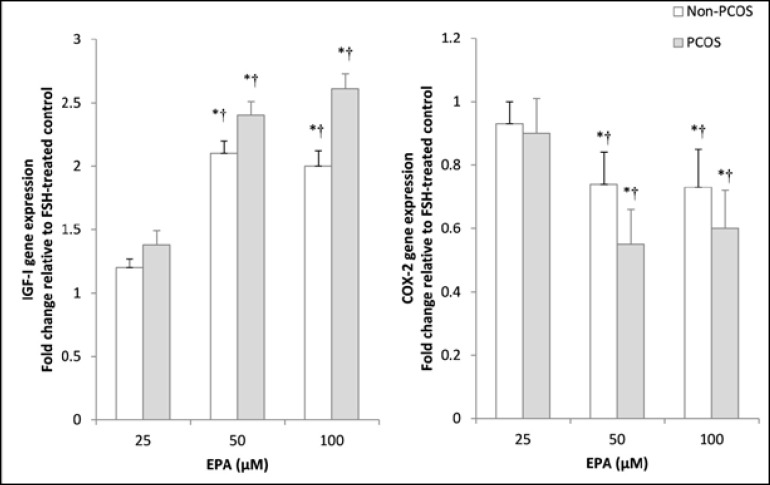
Effect of different doses of eicosapentaenoic acid (EPA) on IGF-1 and COX-2 expression in rFSH-stimulated granulosa cells from non-PCOS women and patients with PCOS. Granulosa cells, after serum starvation, were incubated in 100 ng/mL rFSH alone or in combination with 25 μmol/L, 50 μmol/L or 100 μmol/L EPA for 24h. Cell lysates were prepared and analyzed by real-time PCR for genes expression levels. Expression of IGF-1 (A) and COX-2 (B) in each lysate were normalized to the amount of GAPDH and represented as fold of rFSH-treated control. The mean±SD of three independent experiments with cells pooled from 5 women per group. * p<0.05 vs. rFSH-treated control. † p<0.01 vs. non-PCOS.

## Discussion

The metabolic characteristics of granulosa cells are important in normal maturation of oocytes (22). IGF-1 is critically important in granulosa cells proliferation and follicle selection (9). Although some studies have shown no significant change in IGF-1, the majority of studies have shown that long chain ω-3 PUFA such as EPA and docosahexanoic acid (DHA) up-regulate IGF-1 in different types of cells (23, 24). These effects may be one of the underlying mechanisms for the positive impact of the omega-3 PUFA on reproduction (24). In a similar way, our results demonstrated that there was mRNA expression of IGF-1 and COX-2 in pre-ovulatory human granulosa cells, and that IGF-1 was increased by EPA. This suggests that EPA may elicit important biological responses in granulosa cells via activation of IGF-1.

IGF-1 is a key regulator of follicular differentiation and other reproductive functions (25). It has been shown that the increase in IGF-1 in response to FSH is important for ovulation (26). No specific data is available regarding the possible interaction between IGF-1 and COX-2 in granulosa cells. Increased intrafollicular PGE2 levels are associated with an increased COX-2 expression. PGE2 promote the expansion of the cumulus cells, which is linked to oocyte maturation (27). It has been reported that the expression of IGF-1 mRNA in human ovarian cancer cells is directly related to the expression of COX-2 mRNA (11). Notably, a reciprocal relationship has been shown in which COX-2 stimulates IGF-1 receptor mRNA expression resulting in enhanced IGF-1 induced COX-2 expression in theca cells (28). 

Controversially, findings from an in vivo study showed that COX-2 pathway was associated with an inhibition of the liver IGF-1 biosynthesis and a lower secretion of IGF-1 (12). EPA targets several signaling molecules such as sterol regulatory element binding protein 1c, PPAR receptor type α and retinoic acid receptors, which are potentially involved in the regulation of rFSH-mediated signaling in granulosa cells (29, 30). Future studies about the impact of EPA on granulosa cells should evaluate the functional effects of modulated IGF-1 and COX-2 gene expression on oocyte maturation and fertility. As shown herein and reported previously, FSH induces the expression of IGF-1 and COX-2 (10). 

Co-treatment with EPA and rFSH resulted in enhanced IGF-1 expression both in control and PCOS granulosa cells. However, altered levels of gene expression in PCOS granulosa in response to the combined drug condition was not similar to that observed in control granulosa. In cultured granulosa cells obtained from patients with PCOS, EPA induced a more pronounced effect with rFSH treatment on the mRNA expression level of IGF-1 and COX-2. 

Consistent with these findings, Coffler *et al *have shown that women with PCOS exhibited dose-dependent hyper responsiveness to FSH and increased production of estradiol in granulosa cells (31). The deregulated response of PCOS granulosa cells to gonadotropins has been associated with the arrest of early antral follicle development (32). Although previous research has shown beneficial effect of ω-3 fatty acids on reproduction, this is the first study to examine the effect of EPA on the gene expression of IGF-1 and COX-2 in human granulosa cells. The regulatory effects were simultaneously analyzed by studying the expression in control and PCOS granulosa cells. 

Since the preliminary findings of the present study were derived from cultured granulosa cells, it remained to confirm the in vivo effect of EPA and to further assess the possible mechanism of action of EPA in the treatment of PCOS.

## Conclusion

EPA affect diversely the gene expression of IGF-1 and COX-2 in granulosa cells, which were more pronounced in PCOS compared to control. These findings represent the possible underlying molecular mechanisms for the positive impact of the ω-3 PUFA on reproduction, especially in patients with PCOS.

## Note

***This article extracted from the M.Sc. thesis. (Mina Zaree).***
